# *ATM/RB1* mutations predict shorter overall survival in urothelial cancer

**DOI:** 10.18632/oncotarget.24738

**Published:** 2018-03-30

**Authors:** Ming Yin, Petros Grivas, Hamid Emamekhoo, Prateek Mendiratta, Siraj Ali, JoAnn Hsu, Monali Vasekar, Joseph J. Drabick, Sumanta Pal, Monika Joshi

**Affiliations:** ^1^ Department of Medicine, Division of Hematology-Oncology, Penn State Cancer Institute, Hershey, PA, USA; ^2^ Department of Hematology and Medical Oncology, Cleveland Clinic Taussig Cancer Institute, Cleveland Clinic, Cleveland, OH, USA; ^3^ Department of Medicine, Division of Hematology-Oncology, University of Wisconsin Carbone Cancer Center, WI, USA; ^4^ Foundation Medicine, Cambridge, MA, USA; ^5^ Department of Medical Oncology, City of Hope Comprehensive Cancer Center, Duarte, CA, USA; ^6^ Department of Medicine, Division of Oncology, Ohio State University Comprehensive Cancer Center, Columbus, OH, USA; ^7^ Department of Medicine, Division of Oncology, University of Washington, Seattle, WA, USA

**Keywords:** biomarkers, prognosis, bladder cancer, genomic alterations, next generation sequencing

## Abstract

**Background:**

Mutations of DNA repair genes, e.g. *ATM/RB1*, are frequently found in urothelial cancer (UC) and have been associated with better response to cisplatin-based chemotherapy. Further external validation of the prognostic value of *ATM/RB1* mutations in UC can inform clinical decision making and trial designs.

**Results:**

In the discovery dataset, *ATM/RB1* mutations were present in 24% of patients and were associated with shorter OS (adjusted HR 2.67, 95% CI, 1.45–4.92, *p* = 0.002). There was a higher mutation load in patients carrying *ATM/RB1* mutations (median mutation load: 6.7 versus 5.5 per Mb, *p* = 0.072). In the validation dataset, *ATM/RB1* mutations were present in 22.2% of patients and were non-significantly associated with shorter OS (adjusted HR 1.87, 95% CI, 0.97–3.59, *p* = 0.06) and higher mutation load (median mutation load: 8.1 versus 7.2 per Mb, *p* = 0.126).

**Materials and Methods:**

Exome sequencing data of 130 bladder UC patients from The Cancer Genome Atlas (TCGA) dataset were analyzed as a discovery cohort to determine the prognostic value of *ATM/RB1* mutations. Results were validated in an independent cohort of 81 advanced UC patients. Cox proportional hazard regression analysis was performed to calculate the hazard ratio (HR) and 95% confidence interval (CI) to compare overall survival (OS).

**Conclusions:**

*ATM/RB1* mutations may be a biomarker of poor prognosis in unselected UC patients and may correlate with higher mutational load. Further studies are required to determine factors that can further stratify prognosis and evaluate predictive role of *ATM/RB1* mutation status to immunotherapy and platinum-based chemotherapy.

## INTRODUCTION

Bladder cancer accounts for about 5% of all new cancers in United States, is more common in men than women, and is the most common location of urothelial cancer (UC). It is estimated that about 79,030 new cases of bladder cancer will be diagnosed in 2017 [1]. Most cases are diagnosed as early stage cancer, but those who present with stage IV seem to have poor outcomes. Cisplatin-based chemotherapy has shown efficacy in the muscle invasive (MIBC) localized and metastatic settings [2, 3]. Recently, immunotherapy has changed the treatment paradigm for advanced UC. However, there are still significant number of patients who do not respond to these therapies, hence there is an urgent need to identify new therapeutic targets and potential biomarkers to improve treatment outcomes in these patients. Understanding the genomic landscape of UC has been one of the important mechanisms to identify predictive and prognostic molecular biomarkers. It is well known that defects in DNA repair genes play an important role in tumorigenesis, progression, treatment responses, and outcomes of UC. Somatic mutations of DNA repair genes are frequently found in UC and have been associated with better response to cisplatin-based neoadjuvant chemotherapy. Plimack *et al*. demonstrated that alterations in *ATM*, *RB1*, *FANCC* genes could render the tumor sensitive to cisplatin-based neoadjuvant chemotherapy in MIBC [4]. Similarly, Liu *et al*. showed that the presence of genomic alterations in *ERCC2* may predict response to cisplatin-based neoadjuvant chemotherapy in MIBC [5]. Although these studies suggested that the presence of mutation in DNA repair genes may be an important biomarker predictive of response, the tumor genomic signature in both of these studies were chosen from patients undergoing cisplatin-based chemotherapy in the neoadjuvant setting in MIBC. Recently Teo *et al.* demonstrated that mutations in DNA damage response genes are associated with better prognosis in platinum-treated advanced urothelial cancer patients [6]. However, this study did not include *RB1* mutation and only 4 of 47 patients (9%) had *ATM* mutations. Thus their significance as a prognostic biomarker for UC in general is not very well defined.

In this study, we tried to determine if previously identified prognostic values of *ATM* and *RB1* genes could be applied to different UC patient populations. We had detailed clinical information and tumor sequencing for patients in The Cancer Genome Atlas (TCGA) dataset (publicly available) and we named this the discovery dataset. In addition, we confirmed our findings in a separate dataset, called the validation dataset (CPC), for patients with metastatic UC who had tumor next generation sequencing performed in the context of either routine care or screening evaluation for clinical trials. We assessed association between genetic mutations with survival outcome and tumor mutational load.

## RESULTS

### Patient characteristics

Clinical and pathological characteristics of the patients are shown in Table 1. The median age of TCGA dataset was 69 (range, 34 to 88), while the median age of CPC dataset was 65 (range, 44 to 84). The two datasets shared similarities in several clinicopathological characteristics, including a predominance of male gender, Caucasian race, and a high proportion of ever smokers. However, the two datasets differed significantly in two parameters, tumor stage and site of origin. Only 34.6% (45/130) patients in TCGA dataset were stage IV, while all patients in CPC dataset had stage IV disease at study entry. In addition, TCGA dataset consists of pure bladder cancer, while CPC dataset includes 25.9% non-bladder origin urothelial cancer, such as cancers from renal pelvis and ureter.

**Table 1:** Patient characteristics

Parameters	Discovery (*n* = 130)	Validation (*n* = 81)
**Age (years, range)**	69 (34 – 88)	65 (44 – 84)
**Gender**		
** Male**	98	63
** Female**	32	18
**Race**		
** Non-white**	23	11
** White**	107	70
**Stage**		
** II**	38	
** III**	47	
** IV**	45	81
**Smoking**		
** Never**	20	18
** Ever**	106	63
** Unknown**	4	0
**Site of origin**		
** Bladder**	130	60
** Non-bladder**	0	21

### Frequency and type of *ATM/RB1* mutations

**Figure 1:**
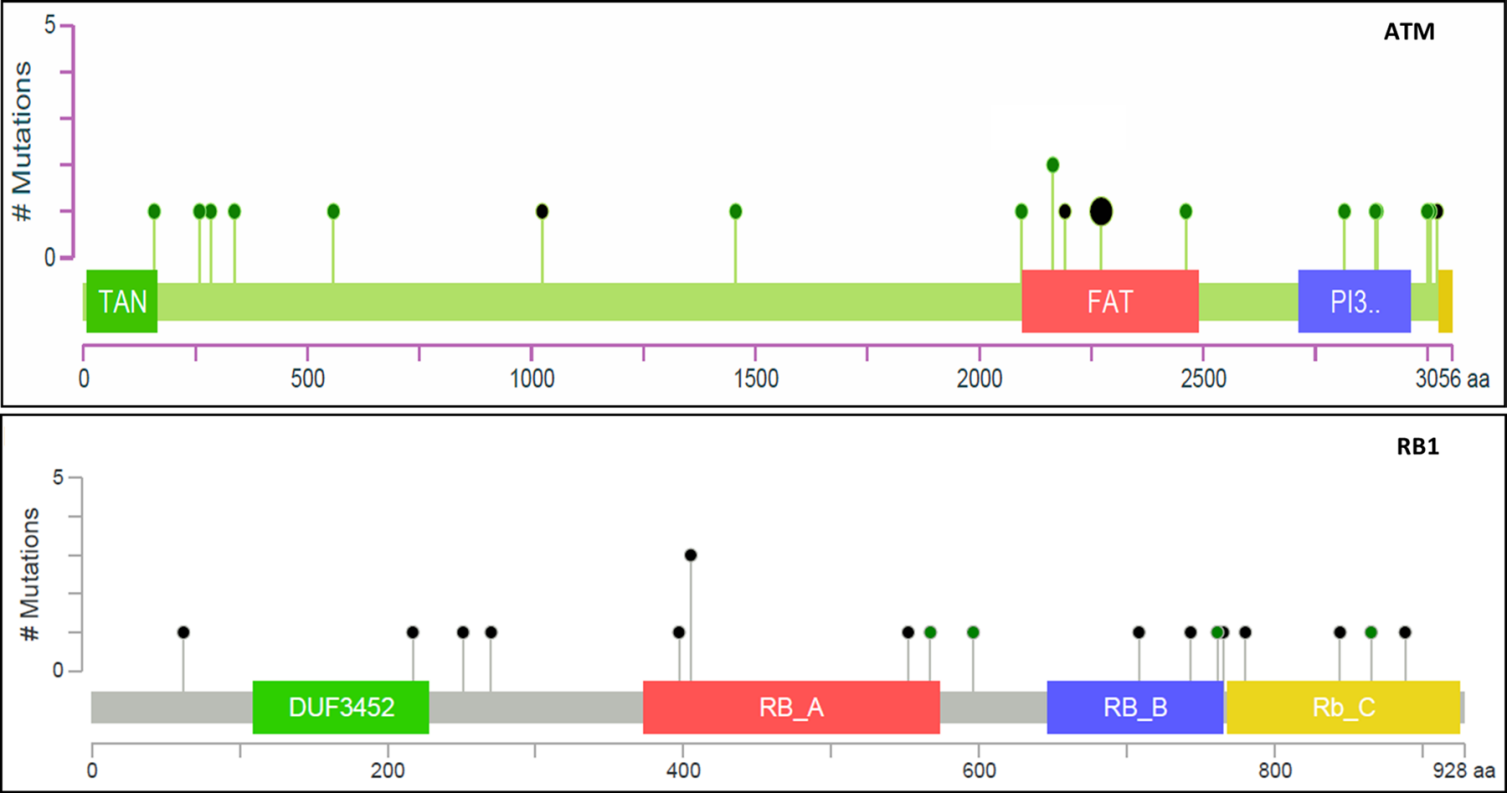
Illustration of missense (green) and truncation (black) mutations in *ATM* and *RB1* genes in TCGA database

Overall, 31 out of 130 patients (24%) had mutations in either *ATM* or *RB1* (*ATM*/*RB1*) genes in the TCGA dataset, while 18 out of 81 patients (22.2%) had mutations in the CPC dataset. *ATM* and *RB1* mutations were not mutually exclusive (*p* = 0.383, Fisher exact test), and could be co-present in one tissue sample. As shown in Table 2, a total of 38 mutations were present in 31 tumor tissue samples in the TCGA dataset, and 28 mutations in 18 samples in the CPC dataset. In the combined dataset, most mutations in *ATM* gene were missense mutations (71%, 22/31), while the majority of mutations in *RB1* gene were truncation mutations (74.3%, 26/35). Figure 1 shows examples of mutations of *ATM* and *RB1* genes in TCGA dataset.

**Table 2:** Types of mutations

	ATM	RB1
**Discovery**		
Missense	15	4
Truncation	4	15
**Validation**		
Missense	7	5
Truncation	5	11

### Associations of *ATM/RB1* mutations with OS

**Figure 2:**
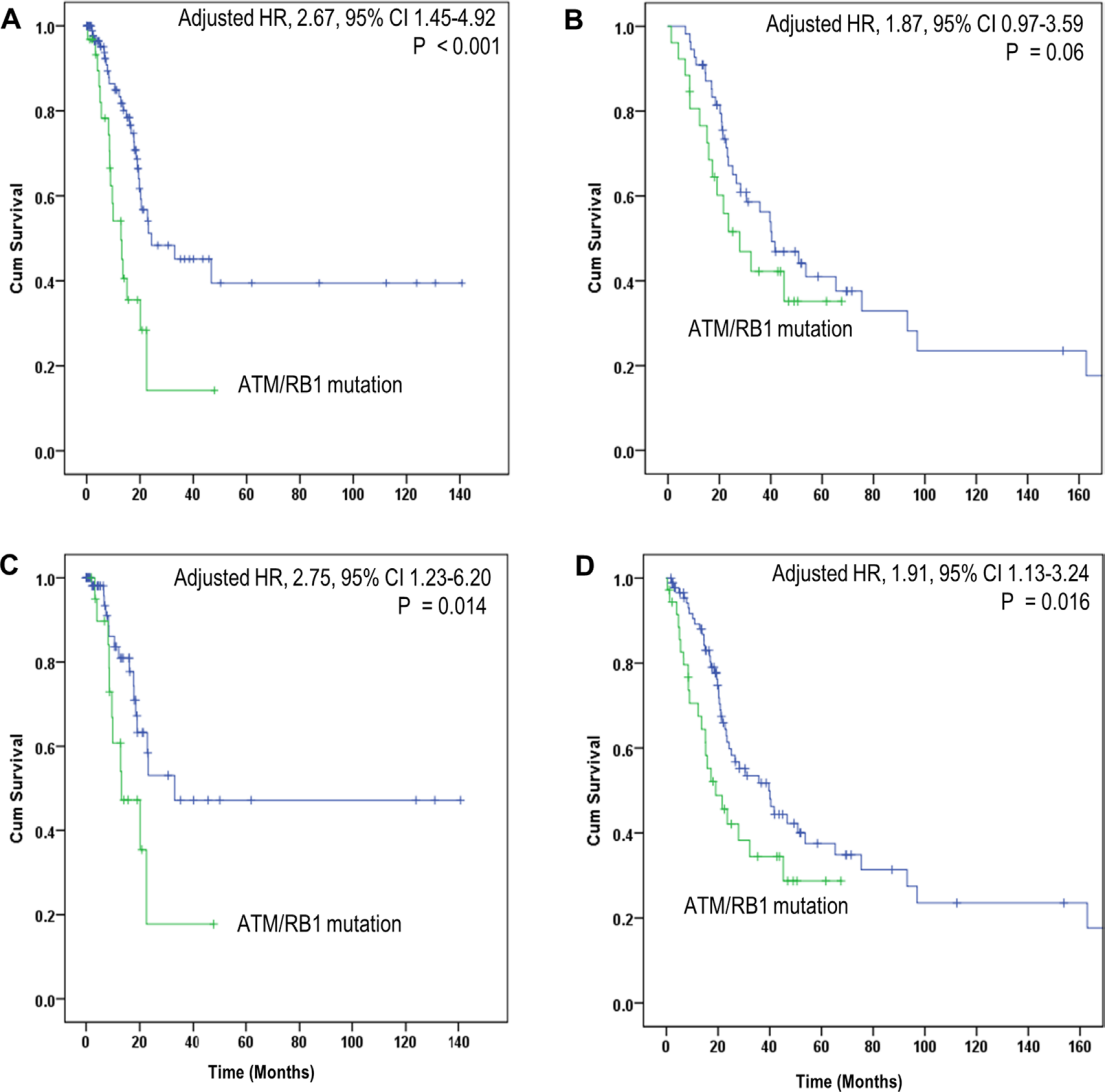
Comparison of OS in patients with mutated and wild type *ATM/RB1* genes (**A**) Discovery dataset; (**B**) Validation dataset; (**C**) Stage II-III subgroup in combined dataset; (**D**) Stage IV subgroup in combined dataset.

The median OS time from cancer diagnosis for TCGA and CPC datasets was 12.8 and 21 months. In the TCGA dataset, *ATM* or *RB1* mutations seemed to be individually associated with shorter OS, while patients with either *ATM* or *RB1* mutations had a significantly shorter OS, compared with those with wild-type *ATM*/*RB1* genes [crude hazard ratio (HR) = 2.76, 95% confidence interval (CI), 1.52–5.02, *p* < 0.001]. In multivariable Cox regression analyses, with adjustment of age, tumor stage, radical cystectomy and smoking history, we found similar results (adjusted HR = 2.67, 95% CI, 1.45–4.92, *p* < 0.001). In the CPC dataset, *ATM*/*RB1* mutations were also associated with shorter OS, although statistical significance was not reached (crude HR = 1.45, 95% CI, 0.78–2.72, *p* = 0.242; adjusted HR = 1.87, 95% CI, 0.97–3.59, *p* = 0.06) (Figure 2). Additional analyses showed *ATM*/*RB1* mutations were associated with non-significant increased hazard ratios in patients with bladder UC and patients who were treated with platinum-based chemotherapy in the CPC dataset (data not shown).

We then performed a pooled analysis by combining TCGA dataset and CPC dataset. In the combined dataset, *ATM*/*RB1* mutations were significantly associated with shorter OS (crude HR = 1.91, 95% CI, 1.24–2.93, *p* = 0.003; adjusted HR = 2.08, 95% CI, 1.35–3.22, *p* < 0.001). In stratified analyses by tumor stage, *ATM*/*RB1* mutations were associated with shorter OS in both stage II/III patients (crude HR = 2.39, 95% CI, 1.09–5.24, *p* = 0.030; adjusted HR = 2.75, 95% CI, 1.23–6.20, *p* = 0.014) and stage IV patients (crude HR = 1.70, 95% CI, 1.01–2.84, *p* = 0.045; adjusted HR = 1.91, 95% CI, 1.13–3.24, *p* = 0.016).

### Comparison of mutational load

**Figure 3:**
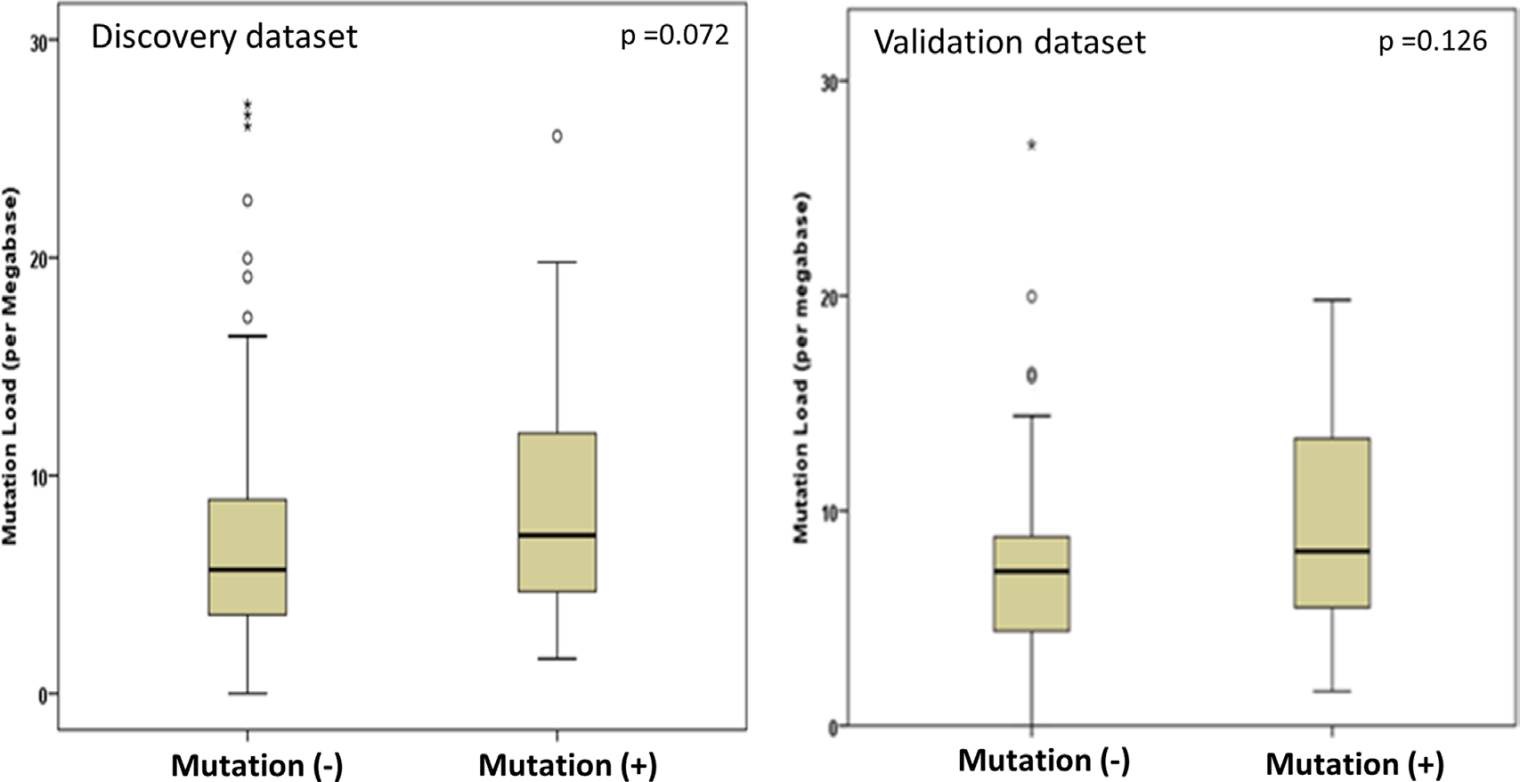
Comparison of mutation load in patients with mutated and wild type *ATM/RB1* genes Left panel, mutation counts per Megabase (Mb) in exome. Right panel, mutation counts per Mb as determined by 315 panel genes.

Tumor tissues harboring *ATM*/*RB1* mutations may have increased mutation rates due to DNA repair defect. The median mutation load was 6.7 versus 5.5 per Mb (*p* = 0.072) in patients with and without *ATM*/*RB1* mutations in TCGA dataset, and was 8.1 versus 7.2 per Mb (*p* = 0.126) in CPC dataset, respectively (Figure 3). In the combined datasets, there was a significant higher mutation load in patients with *ATM*/*RB1* mutations (*p* = 0.02). Interestingly, further analyses by genes showed that only *RB1* mutation in fact was associated a higher mutation load (*p* = 0.004), while *ATM* mutation was not (*p* = 0.88).

## DISCUSSION

Deficiency in DNA damage repair is an early and critical step in tumorigenesis, and plays an important role in tumor progression and treatment response. The current study shows that *ATM*/*RB1* mutations were frequent (around 20%) in UC, appeared to be a biomarker of poor prognosis, and were associated with a higher mutational load.

*ATM* and *RB1* are two well-studied proteins involved in regulation of cell cycle checkpoint signaling and DNA damage response. In general, activation of *ATM* is sparked by DNA double strand breaks (DSB), and subsequently phosphorylates and activates its downstream substrates, including *p*53, BRCA1 and NBS1, which recruits DNA repair complex to initiate both homologous recombination (HR) and non-homologous end joining pathways [7]. *RB1* is a tumor suppressor protein, which inhibits cell cycle progression from G1 to S phase. Recently, there is evidence supporting a direct functional involvement of *RB1* protein in DNA repair by NHEJ [8]. Therefore, loss of function of *ATM* or *RB1* protein may contribute to reduced DSB repair response and uncontrolled cell proliferation, which can lead to increased cancer susceptibility and vulnerability to radiotherapy or cytotoxic agents, e.g. cisplatin or carboplatin.

The role of *ATM* or *RB1* mutations as potential predictive or prognostic biomarkers has been reported in different tumor studies. Teo *et al.* has shown that presence of DNA damage response and repair gene alterations is associated with better clinical outcome in platinum treated patients, but the occurrence of *ATM*/*RB1* mutation in that cohort was very small (approx. 4 out of 47 had *ATM*), so it is hard to compare their findings to ours. A recent study by Plimack *et al.* showed that somatic mutations of *ATM*/*RB1*/*FANCC* genes seemed to confer sensitivity to platinum-based chemotherapy, and were associated with better survival outcome in 34 muscle-invasive bladder cancer (MIBC) patients and a trend towards longer survival in 24 MIBC patients who received cisplatin-based neoadjuvant chemotherapy followed by radical cystectomy [4]. However, our study showed relatively different findings by using two larger datasets of 130 and 81 sample sizes. In the pooled analysis, *ATM*/*RB1* mutations were associated with shorter OS across the study group and in subgroup analysis of stage II-III and stage IV. We do not think our study results conflicted directly with the previous findings because the study population and treatment are not comparable. The study by Plimack *et al.* only included MIBC patients, while our study population was relatively heterogeneous, including both MIBC and stage IV patients. All patients in the study by Plimack *et al.* received platinum-based neoadjuvant chemotherapy followed by surgery with curative intent, while a substantial proportion of our patients had stage IV disease and were treated with palliative intent with or without cisplatin-based chemotherapy. Since treatment information is not available for most TCGA patients, we were unable to perform further analyses in this regard. However, subgroup analysis in stage II/III patients still showed association of *ATM*/*RB1* mutations with poor prognosis. This certainly raises an interesting question whether treatment modalities, regimens and treatment settings, such as neoadjuvant vs. adjuvant, surgery vs. chemoradiation, may contribute to the different findings. In consistency with our results, other studies have shown that *ATM*/*RB1* inactivation were associated with poor prognosis in multiple cancer types, such as leukemia, breast cancer, lung cancer, brain tumor and bladder cancer [9–13]. Further studies are required to determine the underlying reasons for the discrepant findings across studies.

Recently, there is an increased interest in cancer somatic mutation load because it is being considered a potential predictive biomarker of response to immune checkpoint inhibitors across tumor types, including UC [14]. Tumor cells with high mutation load may have more neoantigens, and therefore are more likely to respond to immunotherapy. Defect in DNA repair function can contribute directly to a high mutation load, which has been reported [15]. Our study showed higher mutation load in patients carrying *ATM/RB1* mutations in the combined datasets, suggesting the correlation between DNA repair defect and mutation burden may still be present in sporadic cancers as a result of increased genomic instability through loss of DSB DNA repair. The lack of statistical significance in individual datasets may likely be due to insufficient power. Interestingly, further analyses by genes showed that *RB1* mutation, not *ATM* mutation, was associated with a higher mutation load, which again supported an important role of *RB1* in DNA repair. Although it may be interesting to know the correlation between mutation load and treatment response (e.g. chemotherapy or immunotherapy), we were unable to perform such analyses due to lack of data.

Lastly, there are some limitations of our study. First, due to insufficient treatment information from TCGA dataset, this study focused on the prognostic value of *ATM*/*RB1* mutations instead of their predictive value. Second, we did not include *FANCC* gene in our study because of the extremely low mutation rate (around 1%) in UC which makes it very hard to evaluate in relatively small cohorts. Third, we were only able to use OS, instead of cancer-specific survival (CSS), for endpoint comparison because CSS information is not available for both datasets; however OS is a hard endpoint. Fourth, there may be a selection bias in the CPC dataset, as suggested by longer OS compared with TCGA dataset (21 vs. 12.8 months), as all of our patients were treated at tertiary specialized academic cancer centers. Indeed, most stage IV UC patients died shortly after failing standard chemotherapy and did not have an opportunity for next generation sequencing, while in the CPC dataset patients who lived longer and had a better performance status had a higher chance of being tested by NGS. It is worth mentioning that we did not pursue ascertainment of the mutation functional impact in our study. The functional impact of the mutation on the actual protein status may explain the differential effect on treatment response and outcomes, e.g. deleterious mutations or not. Therefore, we suggest our readers to apply our findings cautiously. In addition, there is increased recognition of the high frequency germline mutations in UC; the relative impact of germline vs. somatic mutations remains unknown. In a study of 53 UC patients, no significant OS difference was noted between patients with and without germline single nucleotide polymorphisms in DNA repair genes [16]. Since homologous recombination deficiency (HRD) score, microsatellite instability, and loss of heterozygosity (LOH) were not reported by Foundation NGS, they were not included in our study and it is hard to comment on the broader genomic instability of each tumor and relevant effect on outcomes. This is a limitation of FoundationOne NGS methodology because comprehensive genomic information can be even more helpful to guide clinical practice than alterations of individual genes; however, FDA recently granted approval to FoundationOne assay [17]. Finally, we acknowledge that TCGA and CPC datasets included UC patients of different characteristics, which may impact the interpretations of our findings. However, stratified analyses by tumor stage in the pooled patient population homogenized patients in subgroups and still showed similar findings.

In summary, our data suggest that *ATM*/*RB1* mutations may be considered a biomarker of poor prognosis in UC and may correlate with higher mutational load. Further studies are required to determine the impact of deleterious mutations, as well as further characteristics that can stratify prognosis based on *ATM*/*RB1* mutation status, and evaluate the potential predictive role of *ATM*/*RB1* mutation status in response to platinum-based chemotherapy and immunotherapy.

## MATERIALS AND METHODS

### Study population

In the discovery dataset, we extracted available clinical and *ATM*/*RB1* somatic mutation data for 130 patients with urothelial bladder cancer from cbioportal for Cancer Genomics (http://www.cbioportal.org) on August 18, 2016, when The Cancer Genome Atlas database website (http://tcga-data.nci.nih.gov) was closed. Although TCGA has now sequenced over 400 bladder cancer patients, it is beyond our ability to obtain those somatic mutation data for further analysis. In the validation dataset (CPC), we included 81patients with metastatic UC from three academic medical centers: (1) City of Hope Comprehensive Cancer Center, (2) Penn State Hershey Cancer Institute, and (3) Cleveland Clinic Taussig Cancer Institute. Those patients had comprehensive genomic sequencing using Foundation One. It is notable to point out that the TCGA data samples were chemotherapy-naïve, muscle-invasive, high-grade urothelial tumors, while 18% of CPC tumor samples were collected after systemic therapy had been administered.

### Genomic profiling

The sequencing methodology of 130 TCGA bladder samples was previously described [18]. For the sequencing methodology of 81 CPC tumor samples, all cases were sequenced with deep coverage across all coding exons from 315 cancer-related genes and 31 genes often related to rearrangement using the commercially available CLIA-approved Foundation One assay. Cases were sequenced to a median depth of 650x. Base substitutions, short insertions, deletions, copy number changes, gene fusions and rearrangements were assessed in a manner akin to previous reports [19]. A comprehensive list of gene alterations included in the Foundation Medicine assay has been reported by Frampton *et al*. [19].

### Statistical analysis

Fisher's exact test was used to determine mutual exclusivity of genes. Clinical outcomes were assessed by overall survival. Univariable Cox regression analysis was performed to calculate crude hazard ratio and 95% confidence interval for death risk, and screen for confounding factors. Multivariable Cox regression was used to control confounding factors. Kaplan-Meier curve was used for cumulative probabilities. We used non-parametric test to compare mutation counts between groups with mutant and wild type *ATM*/*RB1* genes because of outliers. Statistical analysis was performed using SAS 9.1 software (SAS Inc, Chicago, IL). A *p* value of .05 or less was considered statistically significant.
